# Mutational Analysis Gives Insight into Substrate Preferences of a Nucleotidyl Cyclase from *Mycobacterium avium*

**DOI:** 10.1371/journal.pone.0109358

**Published:** 2014-10-31

**Authors:** Wajeed Syed, Melwin Colaςo, Sandra Misquith

**Affiliations:** Department of Chemistry, St. Joseph's College, Bangalore, India; University of Oldenburg, Germany

## Abstract

Mutational, crystallographic and phylogenetic analysis of nucleotidyl cyclases have been used to understand how these enzymes discriminate between substrates. Ma1120, a class III adenylyl cyclase (AC) from *Mycobacterium avium*, was used as a model to study the amino acid residues that determine substrate preference, by systematically replacing ATP specifying residues with those known to specify GTP. This enzyme was found to possess residual guanylyl cyclase (GC) activity at alkaline pH. Replacement of key residues lysine (101) and aspartate (157) with residues conserved across GCs by site directed mutagenesis, led to a marked improvement in GC activity and a decrease in AC activity. This could be correlated to the presence and strength of the hydrogen bond between the second substrate binding residue (157) and the base of the nucleotide triphosphate. This is substantiated by the fact that the pH optimum is highly dependent on the amino acid residues present at positions 101 and 157.

## Introduction

The cyclic nucleotides, cyclic adenosine monophosphate (cAMP) and cyclic guanosine monophosphate (cGMP) are intracellular second messengers with diverse regulatory functions in both unicellular and multicellular organisms [1]. Hence there are an extreme variety and large number of isoforms of these nucleotidyl cyclases [2]. In prokaryotes there are five classes of adenylyl cyclases (I, II, IV, V, VI) that are absent in eukaryotes. Prokaryotes also display an unequalled variety of the universal class III adenylyl cyclases [3]. The abundance of cAMP producing enzymes forms a stark contrast to the presence of only a few putative guanylyl cyclases in prokaryotes [4], [5]. This was subsequently confirmed by sequence alignment studies [6]. Though the functional roles of GCs in prokaryotes are yet to be unraveled, recently Marden et al. and An et al. have identified cyclic GMP dependent signaling pathways in bacteria [7], [8]. Comparison of nucleotidyl cyclases has shown that prokaryotic GCs share a close similarity to bacterial ACs. These bacterial ACs in turn resemble mammalian ACs, as shown by several workers [9]–[14]. Ma1120, an adenylyl cyclase present in *M. avium* shares high sequence similarity with GCs, so this raised the question as to whether Ma1120 could be converted to GC.

Many have tried to use mutational analysis and bioinformatics to understand the evolution of these nucleotidyl cyclases and the conservation of certain amino acid residues at the active sites [3], [15]–[18]. However two groups have reported the conversion of GC to AC by replacing two crucial amino acids at the substrate binding site – namely E to K and C to D [19]–[21].This is probably due to the fact that the crystal structures of mammalian adenylyl cyclases helped to understand theinteractions of K and D with the substrate [22]–[24]. This has not been the case with the guanylyl cyclases where the conservation and interaction of specific residues with GTP is not as clearly defined as in the case of the adenylyl cyclases [5], [25], [26]. The *CYG12* guanylyl cyclase from *Chlamydomonas reinhardtii* contains an E-C pair [25] typical of mammalian guanylyl cyclases while the bacterial Cya2 guanylyl cyclase has an E-G pair [5]. Changing the substrate binding residues has often led to a diminishing of activity rather than a conversion from adenylyl cyclase to guanylyl cyclase [27], [28].

**Figure 1:**
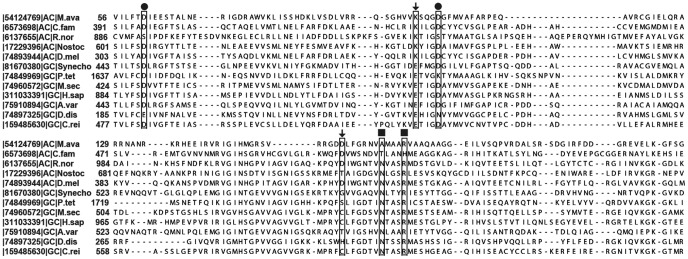
Amino acid sequence alignment of the catalytic region of adenylyl and guanylylcyclases using T-COFFEE web server. First column has the GI accession numbers of proteins available in National Center for Biotechnology Information database followed by type of nucleotidyl cyclase and species names (M.ava: *Mycobacterium avium*; C. fam: *Canis lupus familiaris*; R. nor: *Rattusnorvegicus*; Nostoc: *Nostoc sp. PCC 7120*; D.mel: *Drosophila melanogaster*; Synecho: *Synechocystis sp. PCC 6803*; P. tet: *Paramecium tetraurelia*; M.sec:*Manducasexta*; H.sap: *Homo sapiens*; A.var: *Anabaena variabilis ATCC 29413*; D. dis: *Dictyosteliumdiscoideum*; C. rei: Chlamydomonasreinhardtii). The second column indicates the amino acid position of the domain in the respective sequences. Critical metal binding residues are indicated by•, substrate specifying residues by 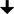 and transition state stabilizing residues are depicted by▪.

Multiple sequence alignment of Ma1120 cyclase domain with representative cyclase domains of ACs and GCs (Fig. 1) shows that the substrate binding residues, lysine (K) and aspartate (D) are conserved in ACs across species. In GCs, glutamate is present instead of K while in place of aspartate, one observes a variety of seemingly unrelated amino acid residues that include cysteine(C), serine(S), threonine(T), histidine(H), alanine(A) or glycine(G).

In this paper we address the question – how do the amino acid residues at the second substrate binding site dictate the nucleotidyl triphosphate preference of the enzyme. For this purpose, Ma1120 having K (101) and D(157) was used as a model to study the consequences of replacing ATP specifying residues with GTP specifying residues. This would help understand how preference for substrates could have evolved.

## Materials and Methods

### Sequence comparison & designing of primers

The clone cya1120 was a kind gift from Prof. S. Visweswariah,IISc, Bangalore. Primers used for mutagenesis (mutagenic primers - Fwd and Rvs complementary to each other) were designed using gene tool and synthesized by Sigma-Aldrich such that the mutation lay in the middle of the oligonucleotide with sufficient flanking residues (minimum of 9–12 bp) to allow a T_m_ close to 78°C (Table 1).

**Table 1:** Sequence of the primers along with their respective T_m_ values.

Sl. No.	Primers	OLIGONUCLEOTIDE SEQUENCE	T_m_
1	Ma1120 Fwd-K101E	5′-TCACGTGGTTGAAAGCCAGGGCGAC-3′	67.10°C
	Ma1120 Rvs-K101E	5′-GCCCTGGCTTTCAACCACGTGACC-3′	66.83°C
2	Ma1120 Fwd-D157C	5′-CCGCGGTGATTGTCTATTCGGCCGCAAC-3′	64.40°C
	Ma1120 Rvs-D157C	5′-GCGGCCGAATAGACAATCACCGCGGCG-3′	64.83°C
3	Ma1120 Fwd-D157G	5′-GCGGTGACGGTCTGTTCGGCCGCAAC-3′	76.23°C
	Ma1120 Rvs-D157G	5′-GCCGAACAGACCGTCACCGCGGCG-3′	75.83°C
4	Ma1120 Fwd-D157T	5′-CGCGGTGACACCCTGTTCGGCCGCAAC-3′	77.95°C
	Ma1120 Rvs-D157T	5′-CCGAACAGGGTGTCACCGCGGCGCAC-3′	77.10°C
5	Ma1120 Fwd-D157H	5′-CGCGGTGACCATCTGTTCGGCCGC-3′	74.09°C
	Ma1120 Rvs-D157H	5′-GCCGAACAGATGGTCACCGCGGCG-3′	74.09°C
6	Ma1120 Fwd-A167Y	5′-GCG ATG GCGTATCGG GTCGCCGCCC-3′	73.66°C
	Ma1120 Rvs-A167Y	5′-GGCGACCCGATACGCCATCGCGACG-3′	72.02°C
7	Ma1120 Fwd-A166N	5′-GTCGCGATGAACGCGCGGGTCGCC-3′	69.90°C
	Ma1120 Rvs-A166N	5′-GACCCGCGCGTTCATCGCGACGTTG-3′	68.74°C

Site directed mutagenesis was carried out by PCR using complementary primers as described by Shenoy *et al.* [28], [29]. The method involved the synthesis of mutant strands using 10–100 ng of the template DNA, 20 pmol of mutagenic primers (Fwd and Rvs), 1x concentration of the thermostable polymerase buffer, 25 mMdNTP's and 2.5 U of *Pfu* turbo in a total reaction volume of 50 µL. The PCR involved a first step at 96°C for 4 minutes, followed by 18 cycles of denaturation for1 minute at 96°C, annealing at a temperature suitable for the primer for 1 minute, and extension time at 68°C for 10 minutes with a final step of extension at 68°C for 20 minutes.After 18 cycles of PCR, 1 µL of the reaction mix was checked on agarose gel. Then the PCR product was digested with *Dpn*I (1 µL) for 8–12 hrs. The *Dpn*I digested PCR product was transformed into DH10B competent *E.coli* cells and clones were selected and then screened [30].

Introduction of the mutation was confirmed by sequencing which was done by MWG (later Eurofinn India). It was then transformed into *E.coli* DH10B cells. Plasmid DNA was isolated from transformed cells. The insertion of the gene was checked by restriction digestion and agarose gel electrophoresis.

### Expression & purification of Ma1120 and its mutants

Ma1120 gene was cloned in pPRO EX-HT-B with the N-terminal histidine tag. This helped in the purification of the expressed protein using affinity chromatography, on a Ni-NTA agarose column. The purity of the protein was checked by SDS-PAGE. The protocol followed was as described by Shenoy et al. and Ketkar et al. [28], [31] with a few modifications. The cell pellet was freeze thawed five times and then 2 mM phenylmethylsulphonylfluoride (PMSF) and 1 mM benzamidine were added. 1 mL of freeze thawed cells was mixed with 1 mL of lysis buffer and sonicated using a VirSonic 50 (Vertis, USA) sonicator for 8 minutes. Sonicated cells were centrifuged at 30,000×g for 45 minutes at 4°C. The supernatant was loaded ontoNi-NTA column. The procedure according to Ketkar et al. [31] was then used for washing and eluting of the column.

### AC and GC assays

Adenylyl cyclase assays were carried out with approximately 500 nM of protein (50 mM MES, HEPES and diethanolamine - a triple buffer system, at appropriate pH), 10 mM NaCl, 5 mM β-mercaptoethanol, 1 mM ATP, 11 mM Mn^2+^& 10% glycerol. The mixture was incubated at 25°C for 10 minutes. The reaction was stopped with 50 mM sodium acetate buffer (pH 4.75) and samples were boiled for 10 minutes. Similarly guanylyl cyclase assays were performed with 500 nM of protein (50 mM triple buffer system, pH 7.5), 10 mM NaCl, 5 mM β-mercaptoethanol, 1 mM GTP, 11 mM Mn^2+^& 10% glycerol. Reaction was done at 37°C. The conditions used were as reported by Ketkar et al. [28], [32], ensuring that the amount of substrate consumed was a fraction of the total substrate present in the reaction mixture as shown in Table S1for ATP and Table S2 for GTP. Amount of cAMP and cGMP produced was determined by radioimmunoassay. All assays included substrate and enzyme blanks as controls.

### AC and GC activity of Ma1120 and its mutants at varied pH

Adenylyl and guanylyl cyclase assays of Ma1120 and its mutants were performed at different pH (5.0, 6.0, 7.0, 7.5, 8.0, 9.0 and 10) conditions using triple buffer (MES, HEPES and diethanolamine) at 50 mM concentration as described above. cAMP and cGMP measurements were carried out by radioimmunoassay.

### Determination of K_m_ and V_max_ of Ma1120 and its mutants

K_m_ and V_max_ of Ma1120and its mutants were determined by measuring the cAMP/cGMP formed by varying the concentrations of substrate at fixed enzyme concentration. AC and GC assays of Ma1120 and its mutants were performed in presence of varied concentrations of substrate (0–2000 µM ATP/GTP), 11 mM Mn^2+^, 50 mM buffer (MES, HEPES and diethanolamine), 10 mM NaCl, 5 mM β-mercaptoethanol and 10% glycerol at pH 8. The enzyme concentration used for the assays was 0.7 µg (500 nM). Radioimmunoassay was used to detect the cAMP/cGMP produced by the enzyme. Data analysis and curve fitting of enzyme kinetics were done using GraphPad Prism software (San Diego, USA).

## Results

### Rationale for preparing mutants, their expression and purification

Sequence comparison of ACs and GCs indicated that while ACs have K as the first substrate binding residue, E was present in GCs as seen in Fig. 1. Therefore, in Ma1120, an adenylyl cyclase, K (101) was replaced by E. Then systematically the second substrate binding residue D (157) was replaced by those residues commonly seen in GCs. As many GCs have C at the corresponding position, the first such mutant had a C instead of D. While several putative GCs have been identified in prokaryotes, the best studied is the one present in cyanobacteria namely Cya2, where there is a G present as the second substrate binding residue [5]. Hence D was mutated to G. As some GCs and ACs have T at the second substrate binding site, the other mutant involved a change from D to T. H has been observed in a GC from *Dictyostelium discoideum*. Hence D (157) was replaced by H [14].

The efficiency of an enzyme is enhanced by the formation of a stable transition state. Most ACs and GCs have an asparagine-arginine (N-R) pair as transition state stabilizing residues [33]. In Ma1120, while R is conserved there is an A (164) instead of N. We asked the question whether a change from A to N would enhance nucleotidyl cyclase activity.

Ma1120 has alanine at position 167 corresponding to a tyrosine residue in Cya2. Rauch et al. [5] showed that this tyrosine residue had no effect on AC activity but greatly enhanced GC activity. Thus a mutation at position 167 was introduced where A was replaced by Y in the double mutant (K101E/D157G) which had the best GC activity.

**Figure 2:**
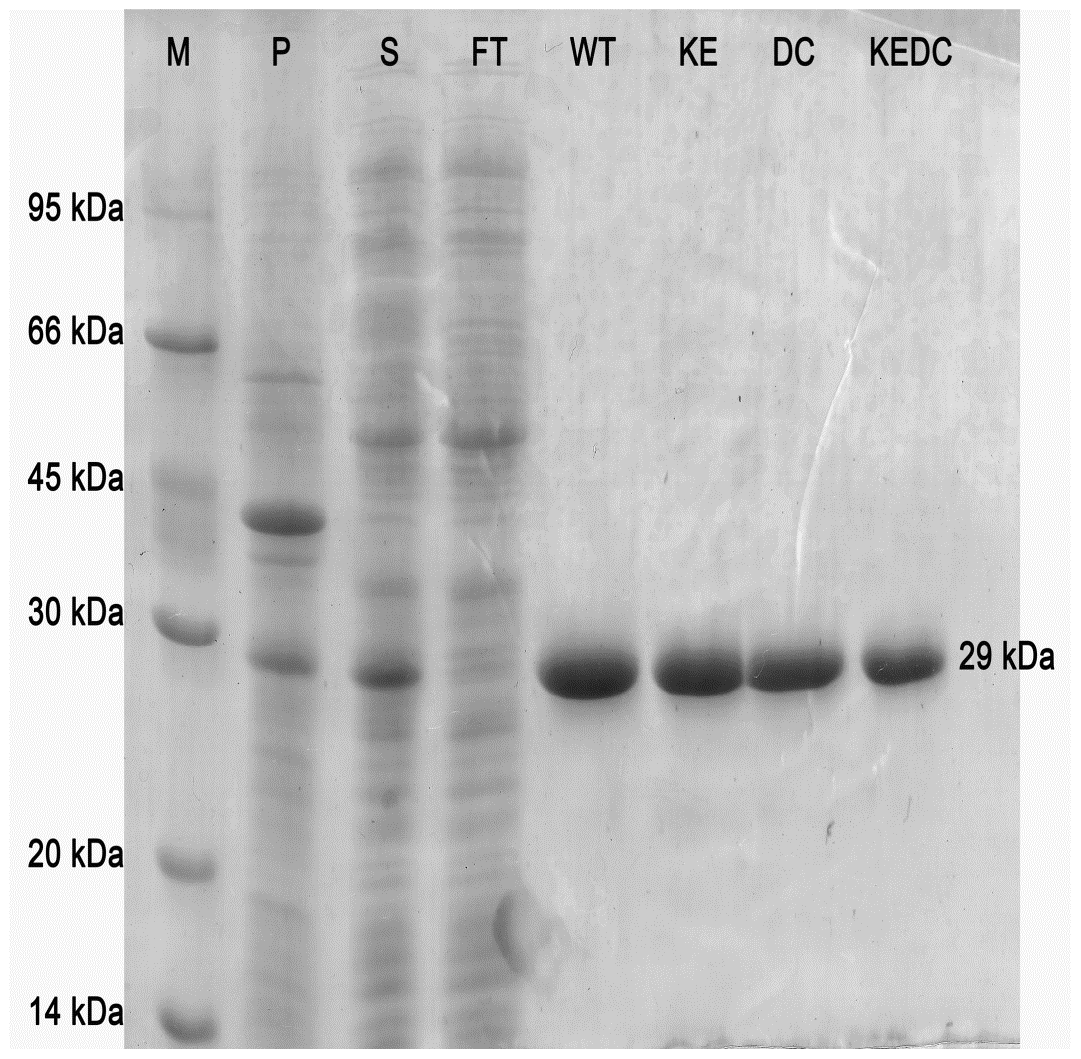
Coomassie stained 15% SDS- polyacrylamide gel showing purified Ma1120 and representative Ma1120 mutant proteins. *M*: Marker, *P*: pellet, *S*: supernatant, *FT*: flowthrough, *WT*: Ma1120, *KE*: K101E, *DC*: D157C and *KEDC*: K101E/D157C.

Ma1120 and the mutant proteins were expressed in *E.coli* BL21DE3 cells and purified using Ni-NTA agarose. The proteins were found to be pure by SDS-PAGE and banded at about 29 kDa. In Fig. 2 the SDS-PAGE profile of representative proteins has been shown and the rest are given in Fig. S1.

### Enzyme activity studies and influence of pH

**Figure 3:**
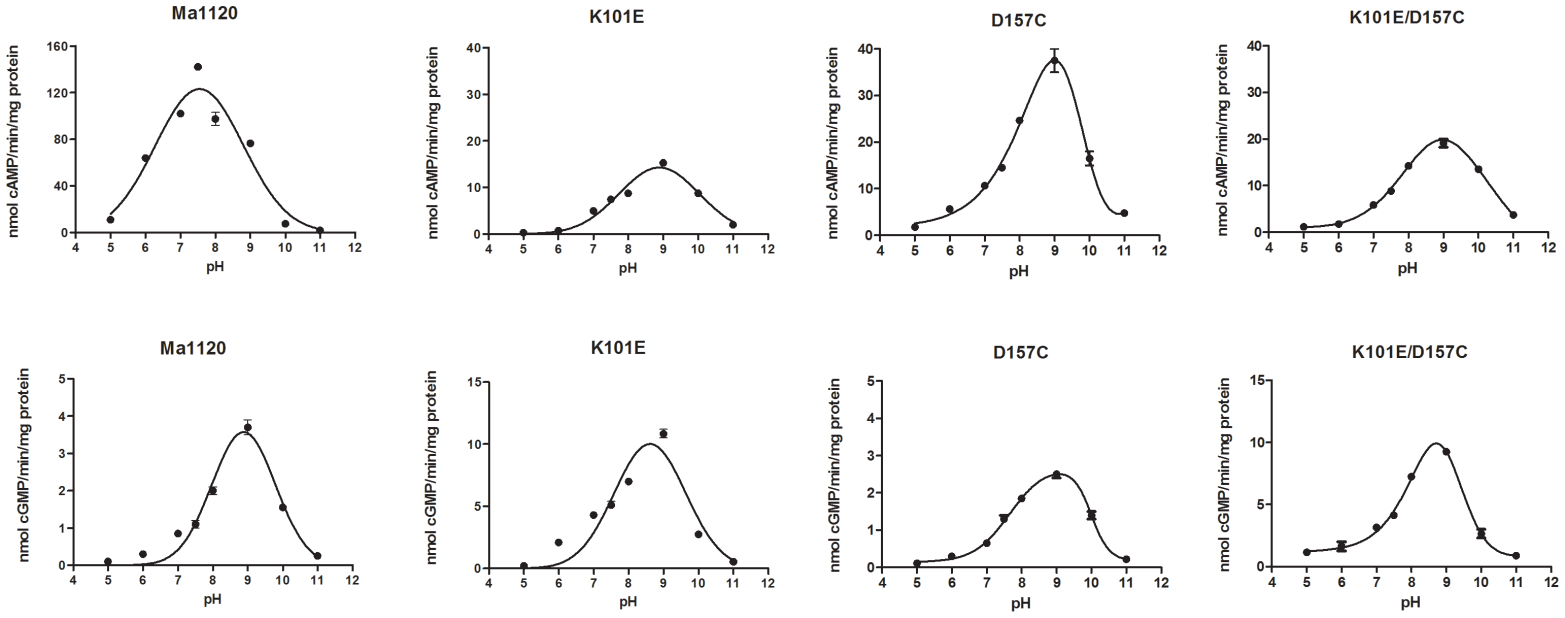
Variation of adenylyl cyclase and guanylyl cyclase activity of Ma1120, K101E, D157C and K101E/D157C with pH.Adenylyl and guanylyl cyclase assays of Ma1120 andits mutants were performed at different pH (5, 6, 7, 7.5, 8, 9, 10 and 11) conditions using triple buffer (MES, HEPES and diethanolamine) at 50 mM concentration and enzyme concentration of 500 nM. cAMP and cGMP measurements were carried out by radioimmunoassay.Mean ±SEM are shown from experiments performed twice with quadruplicates.

AC and GC activity of Ma1120 and some of its mutants was carried out at pH varying from 5 to 11. While the optimum pH of Ma1120 was 7.5 when ATP was the substrate, it was 9.0 for GTP as seen in Fig. 3. For the mutants K101E, D157C and K101E/D157C the pH optimum was found to be pH 9 irrespective of whether the substrate was ATP or GTP. For the other mutants the AC and GC activity was determined at pH 7.5 and 9.0. In all cases the activity was higher at pH 9.0 compared to pH 7.5 (Fig. S2). Thus the activities of the mutants were compared with respect to the original activity possessed by the wild type at pH 7.5 and pH 9.0 (Table 2). Interestingly, the AC activity of the single mutant K101E which was practically abolished at pH 7.5 retained at least 40% of its AC activity at pH 9.0. However GC activity was enhanced by about four-fold at pH 7.5 while there was an 80% increase at pH 9.0.

**Table 2:** Comparison of AC and GC activity of mutants with the wild type at pH 7.5 and 9.0. Mean ± SEM are shown from experiments performed twice with quadruplicates.

Mutants	Percentage decrease in AC activity from wild type at		Percentage increase in GC activity from wild type at	
	pH 7.5	pH 9.0	pH 7.5	pH 9.0
K(101)E	92.1±0.57	62.0±0.71	363.6±7.71	77.9±7.28
D(157)C	90.8±0.71	54.8±0.42	18.2±3.89	5.2±4.31
D(157)T	70.5±0.71	24.7±2.12	15.0±2.26	37.5±1.84
D(157)G	79.0±0.42	37.8±3.54	27.7±8.13	79.2±6.51
A(164)N	90.8±0.28	57.2±6.22	−98.2±1.48	−95.4±0.71
K101E/D157C	94.4±0.49	76.9±3.39	277.3±33.45	285.4±3.39
K101E/D157T	84.4±0.85	53.0±5.73	236.4±22.13	429.2±2.05
K101E/D157G	83.8±0.99	53.0±5.73	413.6±16.15	518.3±27.65
K101E/A164N	99.5±0.14	98.9±0.21	−95.5±1.70	−99.5±1.14
K101E/D157G/A167Y	90.4±1.34	38.8±2.55	533.2±43.27	445.8±51.48

– *sign in the last two columns indicates percentage decrease in GC activity relative to the wild type.*

### Determination of K_m_ and V_max_ values

Since all proteins showed good activity at pH 8.0, kinetic studies were carried out at this pH to investigate the differences in activity of the mutants. Uniform conditions were maintained to understand which amino acid residues interacted with the nucleotidyl triphosphates, leading to an enhancement of GC activity. Saturation curves obtained for both ATP and GTP are shown in fig. S3 and fig.S4 respectively.The double reciprocal curves obtained using GraphPad Prism software gave the K_m_, V_max_ and k_cat_ values which have been listed in Table 3. Though the wild type protein had a higher turnover number (k_cat_) for ATP, its association (K_m_) with ATP was almost 5 times less than it was for GTP.In this system K_m_ is most likely representing a measure of affinity for substrate, therefore a decrease in K_m_could indicate an increase in affinity. A single mutational change at position 101 from K to E resulted in a complete reversal of this observation. The turnover number for ATP by the mutant protein K101E decreased 20 fold compared to the wild type Ma1120 while its interaction with ATP increased 10–12 fold. On the other hand it was more efficient than the wild type in converting GTP to cGMP, though its association with GTP decreased 5–6 fold.

**Table 3:** AC and GC assays of Ma1120 and mutants were conducted at varied concentrations of ATP and GTP ( 0 to 2000 µM) and their k_cat_, K_m_ and k_cat_/K_m_ that were calculated using GraphPad prism software are shown.

Enzyme	ATP				GTP			
	V_max_ (nmol cAMP/min/mg protein)	K_m_ (µM-MnATP)	k_cat_ (s ^−1^)	k_cat_/K_m_ (s ^−1^M ^−1^)x 10^6^	V_max_ (nmol cGMP/min/mg protein)	K_m_ (µM-MnGTP)	k_cat_ (s ^−1^)	k_cat_/K_m_ (s ^−1^M ^−1^) x 10^6^
**Ma1120**	166.4±10.4	173.5±33.1	216±20	1.25	3.4±0.2	36.2±14.7	4.2±0.3	0.11
**K101E**	9.396±0.0984	16.45±0.9701	13.42±0.1406	0.8158	7.364±0.2428	205.7±18.64	10.52±0.3469	0.05
**D157C**	38.4±2.9	76.1±24.7	47.9±3.7	0.628	1.7±0.04	39.7±5.5	2.2±0.05	0.05
**K101E/D157C**	8.455±0.3745	112.6±16.70	12.08±0.5350	0.1073	4.53±0.1686	179.2±19.21	6.375±0.2272	0.038
**D157G**	55.28±0.8762	39.89±2.826	78.97±1.252	1.9797	2.491±0.1191	584.0±56.79	3.559±0.1702	0.006
**K101E/D157G**	28.67±0.3256	21.34±1.104	40.95±0.4651	1.9189	14.26±0.1162	70.55±2.21	20.37±0.1660	0.2887
**D157T**	29.75±0.5932	34.69±3.202	42.50±0.8474	0.8162	1.829±0.0694	199.7±21.02	2.613±0.0992	0.01
**K101E/D157T**	28.03±0.3743	12.67±0.8531	40.04±0.5347	3.16	7.418±0.1150	46.68±3.110	10.60±0.1644	0.2271
**D157H**	26.7±3	140.7±63.9	33.4±4.09	0.327	0.6±0.006	123.4±5.3	0.76±0.007	0.0061
**K101E/D157G/A167Y**	51.93±2.8	289.8±48.41	51.5±3.5	0.224	11.75±0.7	65.02±17.46	14.69±0.87	0.225
**A164N**	78.25±6.0	588.2±50	79.31±9.0	0.13				

All assays were performed at pH 8.

Single mutants created with the second substrate binding site namely D157C, D157G, D157T and D157H showed 3–5 fold lower ability to catalyse the conversion of ATP to cAMP. However the K_m_ value for ATP decreased for all these mutants except for D157H whose association with the substrate was comparable to that of the wild type, though its catalytic activity was reduced 6 fold. On the other hand except for D157C all the other mutants had very low interaction with GTP and were also not efficient in converting GTP to cGMP. In fact D157G had little or no interaction with GTP as its K_m_ value compared to the wild type was 20 fold higher, nonetheless its ability to convert GTP to cGMP decreased 2 fold compared to the wild type. Among the double mutants K101E/D157C, K101E/D157G, K101E/D157T and K101E/D157H, it was found thatK101E/D157G was the most efficient at converting GTP to cGMP, though it was still, more specific for ATP compared to GTP.

The introduction of Y instead of A at position 167 in the double mutant, rendered the molecule impartial to both substrates, that is, the preference of the enzyme for both ATP and GTP (k_cat_/K_m_) was the same. Thus with a triple mutation the protein was equally specific for the two substrates even though it bound ATP to a lesser extent than GTP.

In most class III nucleotidyl cyclases the amino acid residues N and R involved in maintaining the stability of the transition state are highly conserved. In Ma1120, while the R(168) is conserved there is an A instead of an N. Hence the mutants A164N and K101E/A164N were assayed for AC and GC activity. Unexpectedly, in the case of A164N while the AC activity was drastically decreased, the GC activity was totally abolished. In the case of K101E/A164N both AC and GC activity were completely abolished. This could be due to one of two reasons. Either there was a change in orientation of the substrate, in particular the ribose sugar at the active site or a conformational change in the protein.

## Discussion

Our study showed that Ma1120 had residual GC activity despite the presence of the two AC specific substrate binding amino acid residues (K101 and D157). In this study the systematic replacement of ATP specifying amino acid residues to GTP specifying ones, has provided information on how these residues interact with the substrate. As in many other ACs, the first substrate binding residue K probably interacts through H-bonding with the N1 of ATPwhile the second substrate binding amino acid residue, aspartate hydrogen bonds to 6-amino group of ATP [34]. However, when K is mutated to E, this hydrogen bond between the ε-amino group of K and the N1 and the 6-amino group of ATP may no longer be possible. The low K_m_ value could be due to an improper orientation of ATP in the active site, leading to a decrease in turnover number as reflected by the k_cat_ value. On the contrary when GTP is the substrate, E can interact through hydrogen bonding with the 2-amino group of GTP which could be in an orientation conducive for catalysis thus resulting in an increase in catalytic turnover. The adenylyl cyclase:ATP analogue complex crystal structure available in the data base (PDB: 1cjk) [23], shows the possibility of three hydrogen bonds when ATP binds to the enzyme if D is present while only two are possible when D is replaced by C. A similar situation may be occurring in Ma1120, which could explain the decrease in activity of adenylyl cyclase in converting ATP to cAMP when D at position 157 is replaced by C.

The role of hydrogen bonding in determining the strength of binding, orientation and catalytic turnover is corroborated by the influence of pH on the activity of the enzyme as seen by the shift of pH optimum from 7.5 for Ma1120 to 9.0 for the mutants. Strong H-bonding leads to an increase in bindingaffinity but not necessarily enhances its preference for the substrate as seen by the k_cat_/K_m_ values. At pH 8.0 only T (R group pK_a_  = 13.0) and G are protonated species while the pK_a_ values of the side chains of the other residues involved in substrate binding are all below 8.0 and hence at this pH they all couldbe deprotonated. The residues D, E and H having pK_a_ 3.9, 4.3 and 6.0 respectively, are almost completely deprotonated (∼99%) while C (pK_a_ 8.3) is 33% deprotonated. Based on already existing crystal structures of adenylyl and guanylylcyclases available in literature [5], [25], [26], [35]–[37] we suggest that strong H-bonding between the substrate and the substrate binding amino acid residues increases the binding but this is not ideal for catalytic turnover which is why protonated species like K101E/D157G and K101E/D157T show enhanced GC activity where the H-bonds are much weaker. It has been shown that H-bond strength changes depending on the charge acquired by the amino acid residue [38] and that this has far reaching consequences on enzyme catalysis. Introduction of amino acid with deprotonated side chains at the pH studied causes an increase in the length of the H-bond thereby weakening interaction. H-bond strengths are known to vary from 1–2 kJ/mol to 165–180 kJ/mol [39], [40].

The introduction of Y to the double mutant K101E/D157G instead of A at position 167 rendered the molecule impartial to both substrates. i.e. the preference of the enzyme for both ATP and GTP (k_cat_/K_m_) was the same. Thus with a triple mutation the protein was equally specific for the two substrates even though it bound to ATP better than GTP. Cya 2, a GC from synechocystis in which tyrosine is present has been shown to have equal affinity for both substrates. Therefore, in this respect, the triple mutant now resembles Cya2 [5].

In Ma1120 one of the two transition state residues is conserved as in other ACs, however mutation of the other residue from A to the conserved residue N in both the wild type and the K101E mutant lead to a total loss of activity. Thus indicating that it is not necessary that otherwise highly conserved residues are automatically the best for a particular AC and that it has evolved such that the new residue is more suited for its catalytic role.

Overall assessment of the present work with that of others on mutational analysis of nucleotidylcyclases suggests that the microenvironment of the active site governs the binding and catalytic turnover. Kasahara *et al.* saw that the AC activity was abolished when the corresponding K was mutated to E in Cya G, an AC having structural resemblance to GCs [27]. A similar observation was also made in the case of Rv1625 where both K to E and KE/DC mutant resulted in loss of AC activity [28]. Thus it is possible in these cases too that it is not only the hydrogen bonding but the strength of the hydrogen bond that would have dictated the extent of binding and the catalytic turnover of the other systems. Linder (1997) has reviewed the selection process of various ACs and GCs using available crystal structures and docking them with their substrates [34]. The models showing the interaction of the purine residues both in ATP and in GTP with the substrate specifying residues are in agreement with our experimental observations.

Crystal structures of Ma1120 and its mutants will definitely throw light on our understanding of the nature of the interaction with the substrate. All the same this is the first AC to be studied by sequentially replacing second substrate specifying amino acid residues with their GC counterparts. In addition we have shown that, greater the affinity of the nucleotidyl cyclase for the substrate, lower is the catalytic turnover. Hence it is required to study each of the nucleotidyl cyclase on its own merit.

## Supporting Information

Figure S1 SDS-PAGE of Ma1120 mutants. Coomassie stained 15% SDS- polyacrylamide gel showing purified Ma1120 and Ma1120 mutant proteins. ***M***: Marker, ***P***: pellet, ***S***: supernatant, ***FT***: flowthrough, ***DT***: D157T, ***KEDT***: K101E/D157T, ***DG*** **:** D157G, ***KEDG***: K101E/D157G. ***KEDGAY***: K101E/D157G/A167Y, ***DH***: D157H, **AN**: A164N and ***KEAN***: K101E/A164N.
(DOCX)

Figure S2 AC and GC activity of Ma1120 (WT) and mutant proteins at pH 7.5 and pH 9.0. Adenylyl cyclase and guanylyl cyclase activity assays were performed at different pH 7.5 and 9.0 using triple buffer (MES, HEPES and diethanolamine) at 50 mM concentration and enzyme concentration of 500 nM. cAMP and cGMP measurements were carried out by radioimmunoassay. Mean ±SEM are shown from experiments performed twice with quadruplicates. ***KE***: K101E, ***DC***: D157C, ***KEDC***: K101E/D157C, ***DT***: D157T, ***KEDT***: K101E/D157T, ***DG*** **:** D157G, ***KEDG***: K101E/D157G, ***KEDGAY***: K101E/D157G/A167Y, ***DH***: D157H, **AN**: A164N and ***KEAN***: K101E/A164N.
(DOCX)

Figure S3 Kinetic analysis of Ma1120 and its mutants with respect to MnATP. Ma1120-WT and mutants (∼500 nM) were assayed by varying the concentrations of MnATP and a fixed excess of 10 mM free Mn^2+^. Mean ±SEM are shown from experiments performed twice with quadruplicate replicates.
(DOCX)

Figure S4 Kinetic analysis of Ma1120-WT and its mutants with respect to MnGTP. Ma1120-WT and mutants (∼500 nM) were assayed by varying the concentrations of MnGTP and a fixed excess of 10 mM free Mn^2+^. Mean ±SEM are shown from experiments performed twice with quadruplicate replicates.
(DOCX)

Table S1 Amount of cAMP formed from 1 mmole of substrate at fixed enzyme concentration. Assays were carried out with approximately 500 nM of protein (50 mM MES, HEPES and diethanolamine - a triple buffer system, at appropriate pH), 10 mM NaCl, 5 mM β-mercaptoethanol, 1 mM ATP, 11 mM Mn^2+^ & 10% glycerol. The mixture was incubated at 25°C for 10 minutes. The reaction was stopped with 50 mM sodium acetate buffer (pH 4.75) and samples were boiled for 10 minutes. Radioimmunoassay was used to detect the cAMP produced by the enzyme. cAMP formed is expressed in nmoles. % of product per substrate is also shown.
(DOCX)

Table S2 Amount of cGMP formed from 1 mmole of substrate at fixed enzyme concentration. Assays were carried out with approximately 500 nM of protein (50 mM MES, HEPES and diethanolamine - a triple buffer system, at appropriate pH), 10 mM NaCl, 5 mM β-mercaptoethanol, 1 mM GTP, 11 mM Mn^2+^ & 10% glycerol. The mixture was incubated at 25°C for 10 minutes. The reaction was stopped with 50 mM sodium acetate buffer (pH 4.75) and samples were boiled for 10 minutes. Radioimmunoassay was used to detect the cGMP produced by the enzyme. cGMP formed is expressed in nmoles. % of product per substrate is also shown.
(DOCX)
