# Supplementary material for: Mutational Analysis Gives Insight into Substrate Preferences of a Nucleotidyl Cyclase from Mycobacterium avium
Source: PLoS One. 2014 Oct 31;9(10):e109358. doi: 10.1371/journal.pone.0109358 (PMC4215837; doi:10.1371/journal.pone.0109358)

**Fig. S1: SDS-PAGE of Ma1120 mutants**. Coomassie stained 15% SDS- polyacrylamide gel showing purified Ma1120 and Ma1120 mutant proteins. ***M***: Marker, ***P***: pellet, ***S***: supernatant, ***FT***: flowthrough, ***DT***: D157T, ***KEDT***: K101E/D157T, ***DG*:** D157G, ***KEDG***: K101E/D157G. ***KEDGAY***: K101E/D157G/A167Y, ***DH***: D157H, **AN**: A164N and ***KEAN***: K101E/A164N.


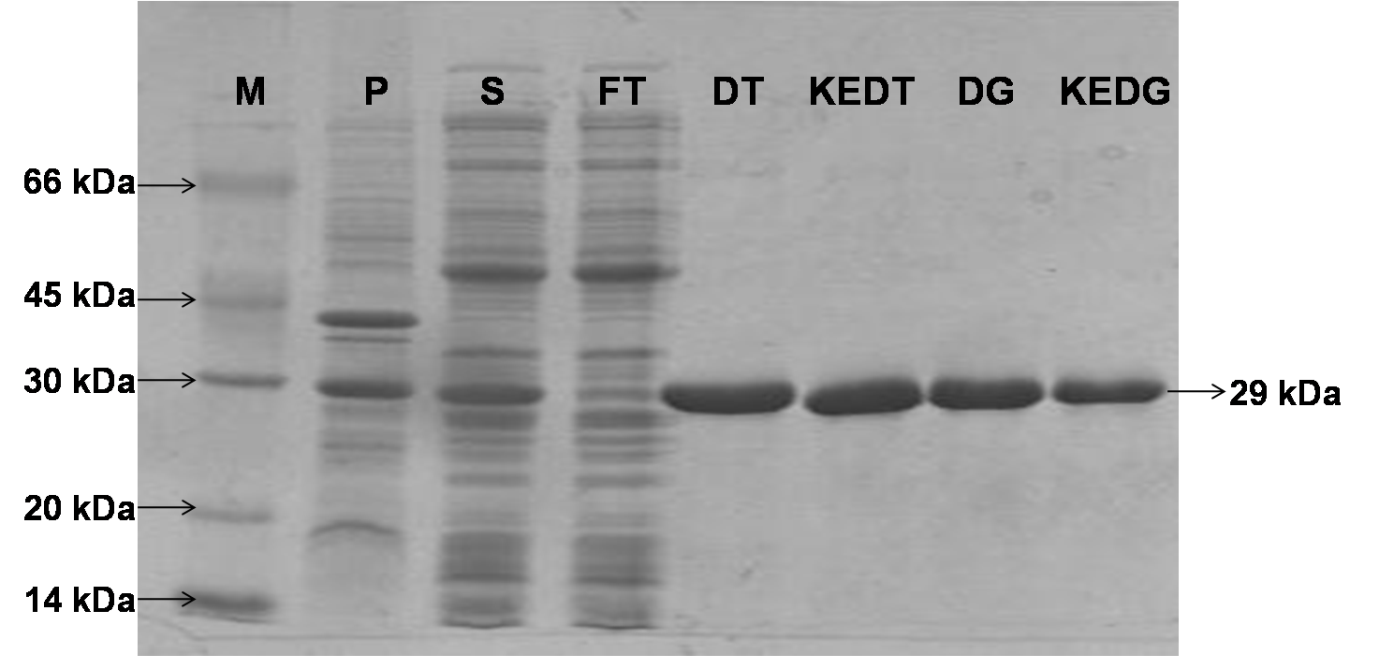


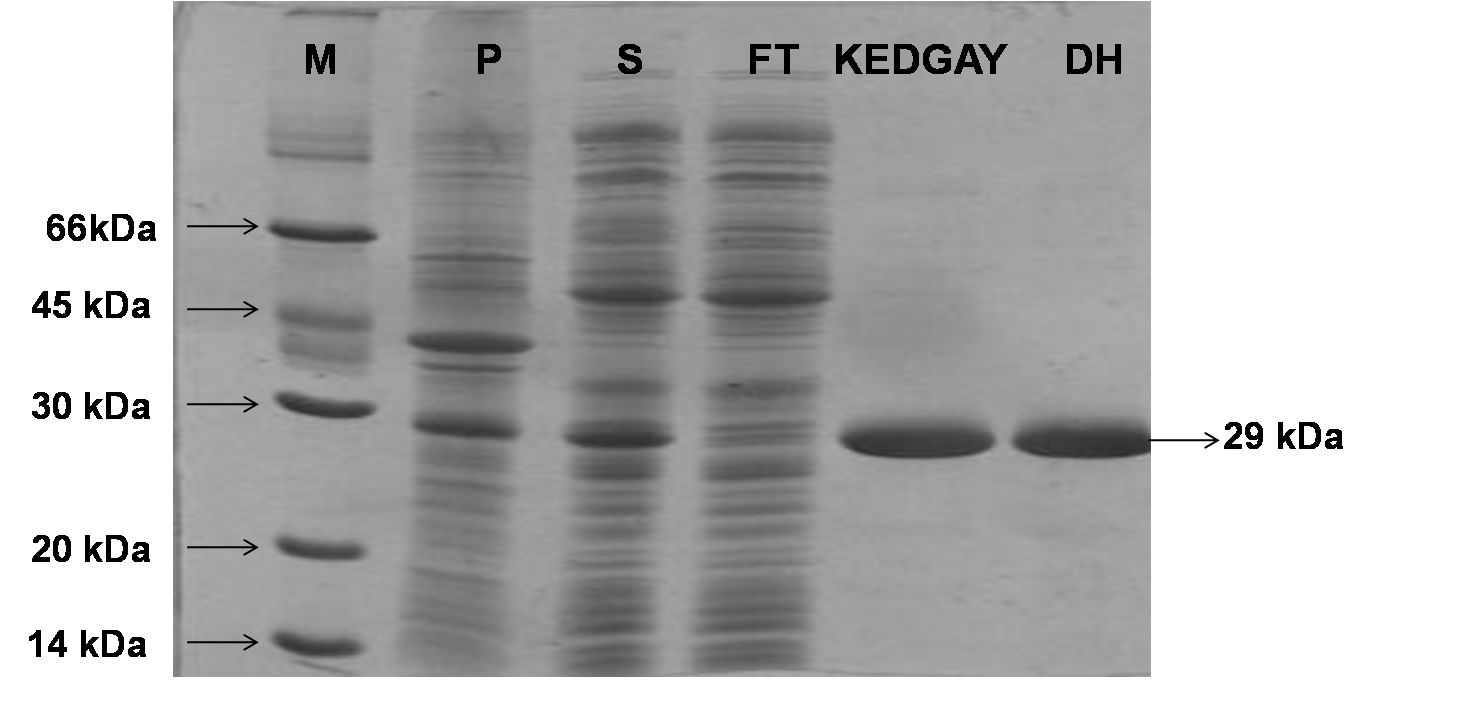


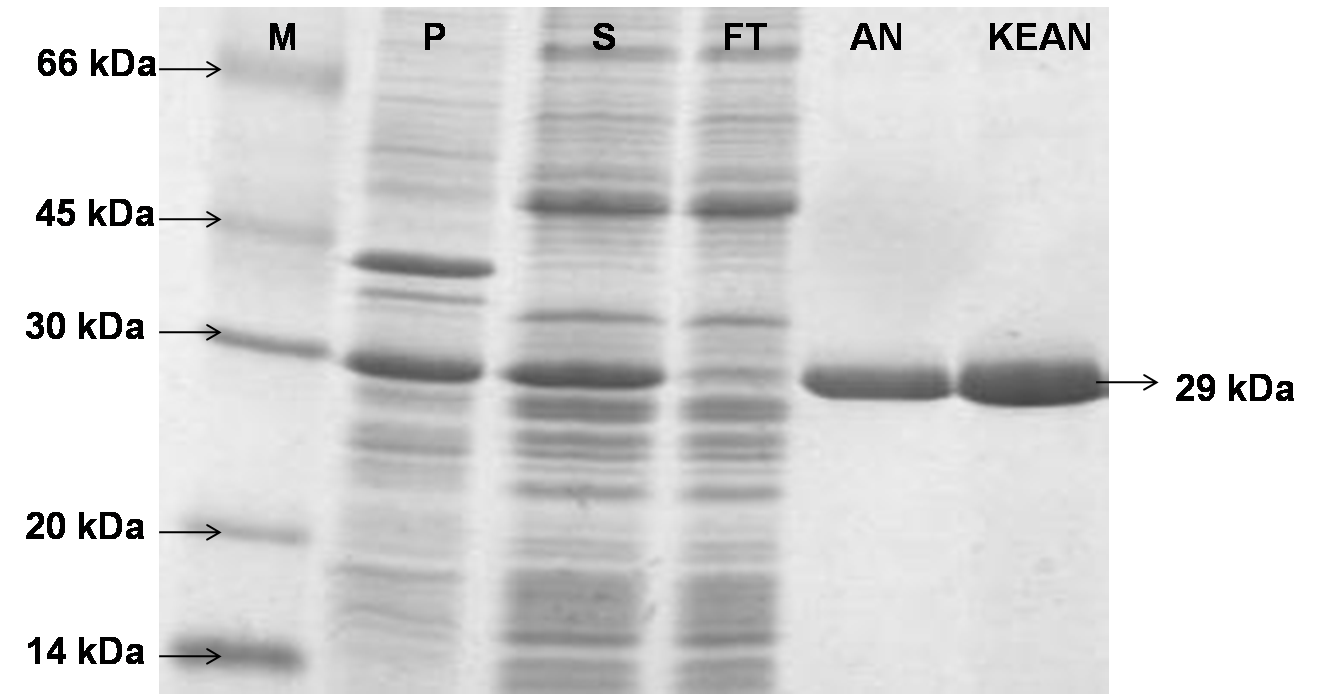

Supplement: Figure S1 — SDS-PAGE of Ma1120 mutants. Coomassie stained 15% SDS- polyacrylamide gel showing purified Ma1120 and Ma1120 mutant proteins. M: Marker, P: pellet, S: supernatant, FT: flowthrough, DT: D157T, KEDT: K101E/D157T, DG : D157G, KEDG: K101E/D157G. KEDGAY: K101E/D157G/A167Y, DH: D157H, AN: A164N and KEAN: K101E/A164N. (DOCX) [file pone.0109358.s001.docx]
