# Supplementary material for: Mutational Analysis Gives Insight into Substrate Preferences of a Nucleotidyl Cyclase from Mycobacterium avium
Source: PLoS One. 2014 Oct 31;9(10):e109358. doi: 10.1371/journal.pone.0109358 (PMC4215837; doi:10.1371/journal.pone.0109358)

**Fig. S2**: **AC and GC activity of Ma1120 (WT) and mutant proteins at pH 7.5 and pH 9.0.** Adenylyl cyclase and guanylyl cyclase activity assays were performed at different pH 7.5 and 9.0 using triple buffer (MES, HEPES and diethanolamine) at 50 mM concentration and enzyme concentration of 500 nM. cAMP and cGMP measurements were carried out by radioimmunoassay. Mean ±SEM are shown from experiments performed twice with quadruplicates. ***KE***: K101E, ***DC***: D157C, ***KEDC***: K101E/D157C, ***DT***: D157T, ***KEDT***: K101E/D157T, ***DG*:** D157G, ***KEDG***: K101E/D157G, ***KEDGAY***: K101E/D157G/A167Y, ***DH***: D157H, **AN**: A164N and ***KEAN***: K101E/A164N.


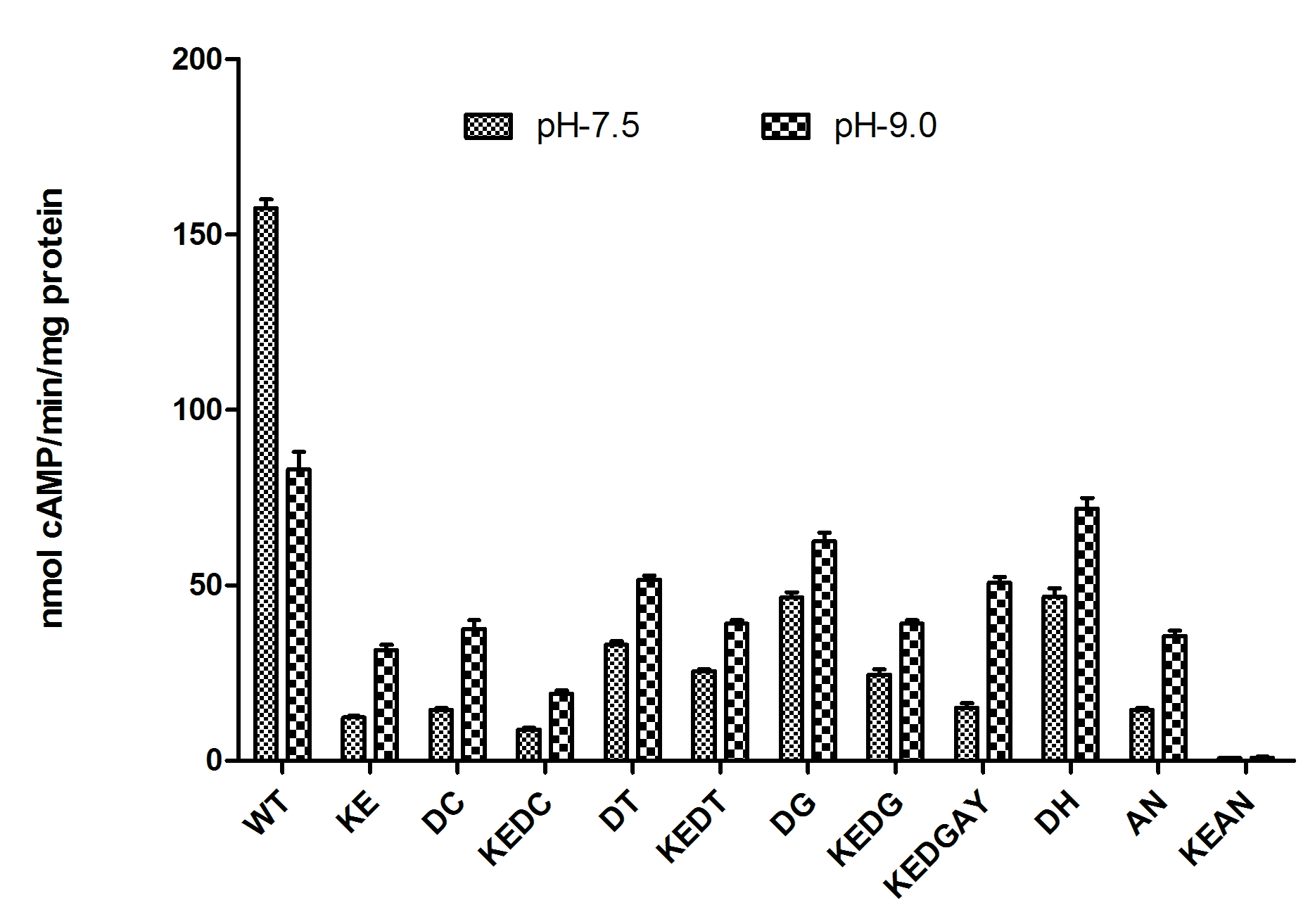


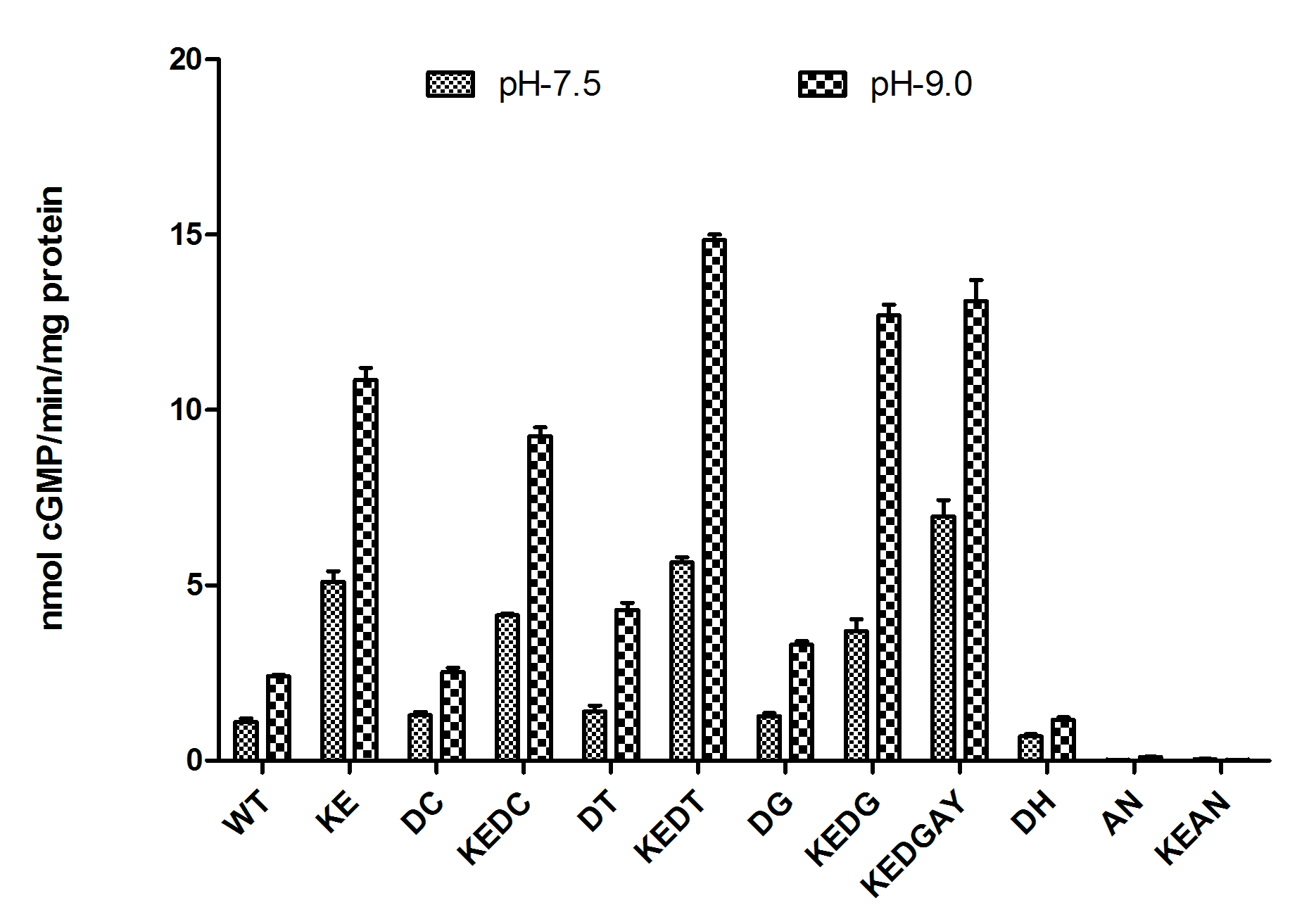

Supplement: Figure S2 — AC and GC activity of Ma1120 (WT) and mutant proteins at pH 7.5 and pH 9.0. Adenylyl cyclase and guanylyl cyclase activity assays were performed at different pH 7.5 and 9.0 using triple buffer (MES, HEPES and diethanolamine) at 50 mM concentration and enzyme concentration of 500 nM. cAMP and cGMP measurements were carried out by radioimmunoassay. Mean ±SEM are shown from experiments performed twice with quadruplicates. KE: K101E, DC: D157C, KEDC: K101E/D157C, DT: D157T, KEDT: K101E/D157T, DG : D157G, KEDG: K101E/D157G, KEDGAY: K101E/D157G/A167Y, DH: D157H, AN: A164N and KEAN: K101E/A164N. (DOCX) [file pone.0109358.s002.docx]
