# Supplementary material for: Mutational Analysis Gives Insight into Substrate Preferences of a Nucleotidyl Cyclase from Mycobacterium avium
Source: PLoS One. 2014 Oct 31;9(10):e109358. doi: 10.1371/journal.pone.0109358 (PMC4215837; doi:10.1371/journal.pone.0109358)

**Fig. S3**: **Kinetic analysis of Ma1120 and its mutants with respect to MnATP.** Ma1120-WT and mutants (~500 nM) were assayed by varying the concentrations of MnATP and a fixed excess of 10 mM free Mn^2+^. Mean ±SEM are shown from experiments performed twice with quadruplicate replicates.


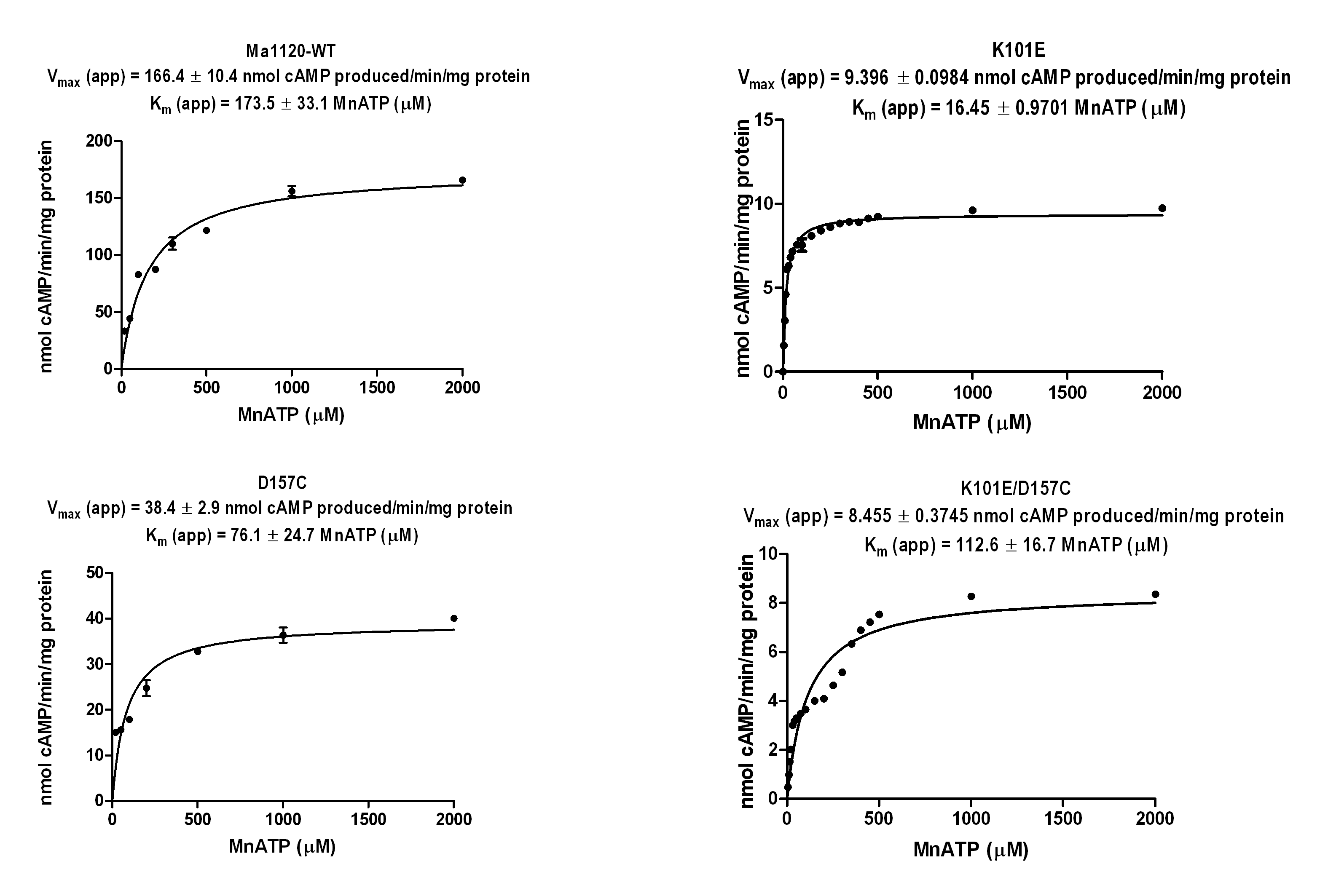


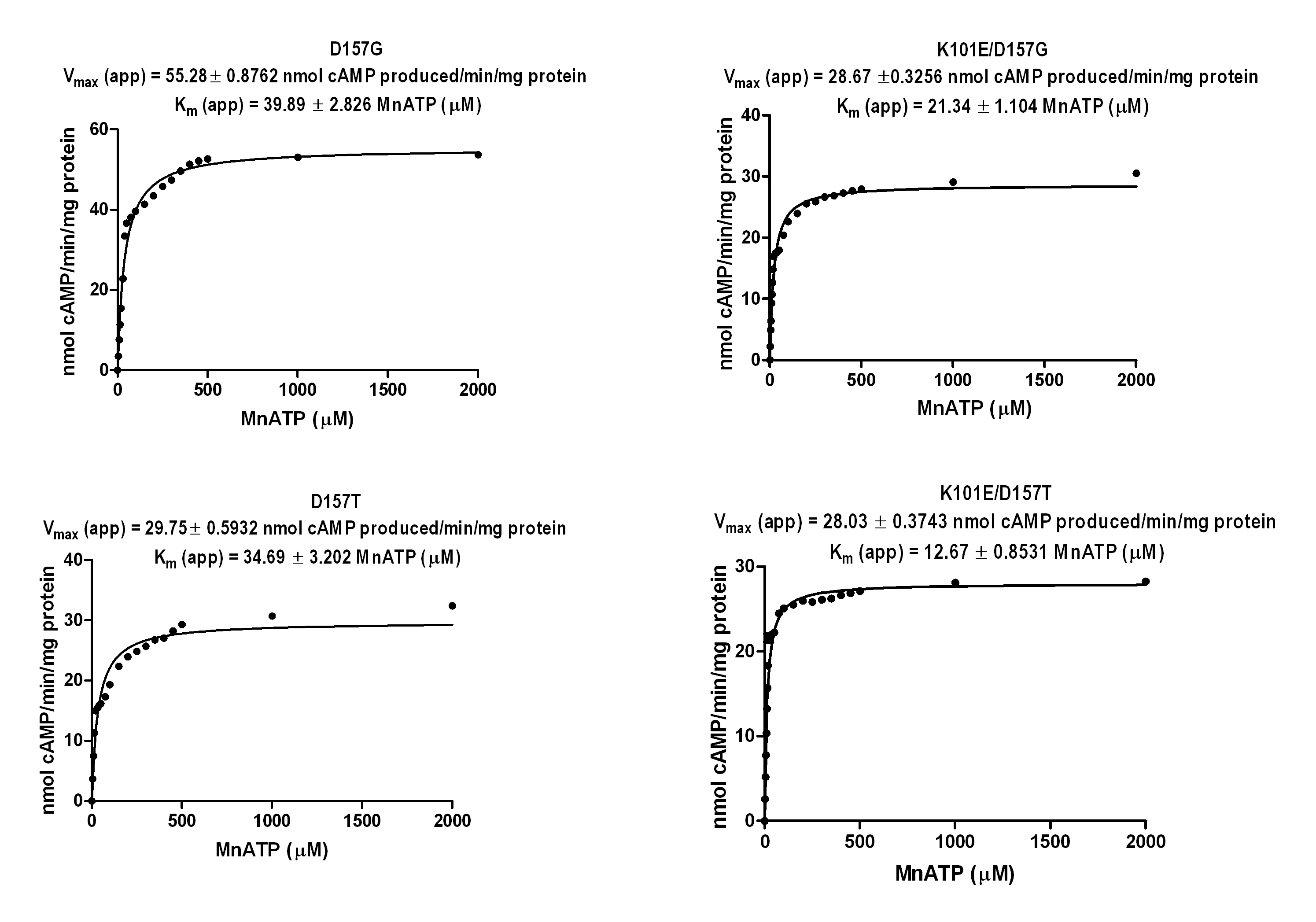


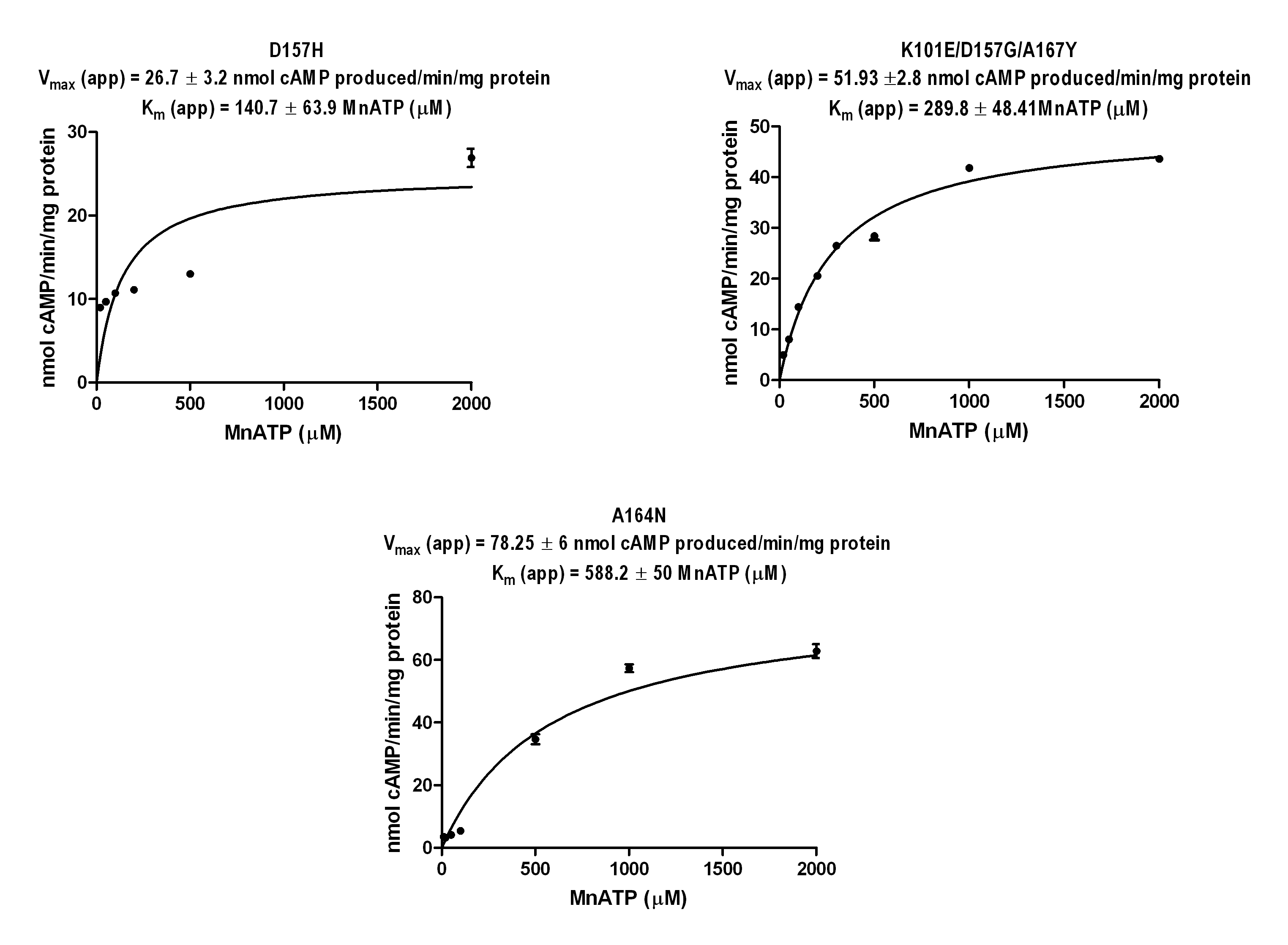

Supplement: Figure S3 — Kinetic analysis of Ma1120 and its mutants with respect to MnATP. Ma1120-WT and mutants (∼500 nM) were assayed by varying the concentrations of MnATP and a fixed excess of 10 mM free Mn2+. Mean ±SEM are shown from experiments performed twice with quadruplicate replicates. (DOCX) [file pone.0109358.s003.docx]
