# Supplementary material for: Mutational Analysis Gives Insight into Substrate Preferences of a Nucleotidyl Cyclase from Mycobacterium avium
Source: PLoS One. 2014 Oct 31;9(10):e109358. doi: 10.1371/journal.pone.0109358 (PMC4215837; doi:10.1371/journal.pone.0109358)
Supplement: Table S1 — Amount of cAMP formed from 1 mmole of substrate at fixed enzyme concentration. Assays were carried out with approximately 500 nM of protein (50 mM MES, HEPES and diethanolamine - a triple buffer system, at appropriate pH), 10 mM NaCl, 5 mM β-mercaptoethanol, 1 mM ATP, 11 mM Mn2+ & 10% glycerol. The mixture was incubated at 25°C for 10 minutes. The reaction was stopped with 50 mM sodium acetate buffer (pH 4.75) and samples were boiled for 10 minutes. Radioimmunoassay was used to detect the cAMP produced by the enzyme. cAMP formed is expressed in nmoles. % of product per substrate is also shown. (DOCX) [file pone.0109358.s005.docx]

**Table S1:** **Amount of cAMP formed from 1 mmole of substrate at fixed enzyme concentration.** Assays were carried out with approximately 500 nM of protein (50 mM MES, HEPES and diethanolamine - a triple buffer system, at appropriate pH), 10 mM NaCl, 5 mM β-mercaptoethanol, 1mM ATP, 11mM Mn^2+^ & 10% glycerol. The mixture was incubated at 25°C for 10 minutes. The reaction was stopped with 50 mM sodium acetate buffer (pH 4.75) and samples were boiled for 10 minutes. Radioimmunoassay was used to detect the cAMP produced by the enzyme. cAMP formed is expressed in nmoles. % of product per substrate is also shown.

| Protein | nmol cAMP/min/mg protein produced (pH-7.5) | % cAMP produced/ substrate used for the assay | nmol cAMP/min/mg protein produced (pH-9) | % cAMP produced/  substrate used for the assay |
| --- | --- | --- | --- | --- |
| WT | 155 | 0.0155 | 78 | 0.0078 |
| KE | 12.8 | 0.00128 | 30 | 0.003 |
| DC | 15 | 0.0015 | 35 | 0.0035 |
| KEDC | 9.34 | 0.00093 | 20 | 0.002 |
| DT | 32 | 0.0032 | 50.6 | 0.005 |
| KEDT | 26 | 0.0026 | 40 | 0.004 |
| DG | 45 | 0.0045 | 60 | 0.006 |
| KEDG | 23 | 0.0023 | 40 | 0.004 |
| KEDGAY | 16.49 | 0.0016 | 49.18 | 0.0049 |
| DH | 49.14 | 0.0049 | 74.98 | 0.0074 |
| AN | 14 | 0.0014 | 35 | 0.0035 |
| KEAN | 0.75 | 0.00075 | 0.9 | 0.0009 |
