# Supplementary material for: Mutational Analysis Gives Insight into Substrate Preferences of a Nucleotidyl Cyclase from Mycobacterium avium
Source: PLoS One. 2014 Oct 31;9(10):e109358. doi: 10.1371/journal.pone.0109358 (PMC4215837; doi:10.1371/journal.pone.0109358)
Supplement: Table S2 — Amount of cGMP formed from 1 mmole of substrate at fixed enzyme concentration. Assays were carried out with approximately 500 nM of protein (50 mM MES, HEPES and diethanolamine - a triple buffer system, at appropriate pH), 10 mM NaCl, 5 mM β-mercaptoethanol, 1 mM GTP, 11 mM Mn2+ & 10% glycerol. The mixture was incubated at 25°C for 10 minutes. The reaction was stopped with 50 mM sodium acetate buffer (pH 4.75) and samples were boiled for 10 minutes. Radioimmunoassay was used to detect the cGMP produced by the enzyme. cGMP formed is expressed in nmoles. % of product per substrate is also shown. (DOCX) [file pone.0109358.s006.docx]

**Table S2:** **Amount of cGMP formed from 1 mmole of substrate at fixed enzyme concentration.** Assays were carried out with approximately 500 nM of protein (50 mM MES, HEPES and diethanolamine - a triple buffer system, at appropriate pH), 10 mM NaCl, 5 mM β-mercaptoethanol, 1mM GTP, 11mM Mn^2+^ & 10% glycerol. The mixture was incubated at 25°C for 10 minutes. The reaction was stopped with 50 mM sodium acetate buffer (pH 4.75) and samples were boiled for 10 minutes. Radioimmunoassay was used to detect the cGMP produced by the enzyme. cGMP formed is expressed in nmoles. % of product per substrate is also shown.

| Protein | nmol cGMP/min/mg protein produced  (pH-7.5) | % cGMP produced/  substrate used for the assay | nmol cGMP/min/mg protein produced  (pH-9) | % cGMP produced/  substrate used for the assay |
| --- | --- | --- | --- | --- |
| WT | 1.00 | 0.0001 | 2.35 | 0.00023 |
| KE | 4.80 | 0.00048 | 10.50 | 0.0010 |
| DC | 1.40 | 0.00014 | 2.40 | 0.00024 |
| KEDC | 4.20 | 0.00042 | 9.00 | 0.0009 |
| DT | 1.23 | 0.00012 | 4.10 | 0.00041 |
| KEDT | 5.50 | 0.00055 | 15.00 | 0.0015 |
| DG | 1.35 | 0.000135 | 3.20 | 0.00032 |
| KEDG | 3.36 | 0.00033 | 12.40 | 0.0012 |
| KEDGAY | 7.43 | 0.00074 | 13.70 | 0.0013 |
| DH | 0.75 | 0.000075 | 1.10 | 0.00011 |
| AN | 0.02 | 0.000002 | 0.1 | 0.00001 |
| KEAN | 0.05 | 0.000005 | 0.01 | 0.000001 |
|  |  |  |  |  |
